# Systematic review of infant and young child complementary feeding practices in South Asian families: the Pakistan perspective

**DOI:** 10.1017/S1368980017002956

**Published:** 2017-11-20

**Authors:** Logan Manikam, Anika Sharmila, Abina Dharmaratnam, Emma C Alexander, Jia Ying Kuah, Ankita Prasad, Sonia Ahmed, Raghu Lingam, Monica Lakhanpaul

**Affiliations:** 1 Population, Policy & Practice, UCL Great Ormond Street Institute of Child Health, 30 Guilford Street, London WC1N 1EH, UK; 2 King’s College Hospital, King’s College Hospital NHS Foundation Trust, London, UK; 3 Leicester Medical School, University of Leicester, Leicester, UK; 4 Institute of Health & Society, Newcastle University, Newcastle upon Tyne, UK

**Keywords:** Infant, Diet, Child, Nutrition, Complementary feeding, Pakistan

## Abstract

**Objective:**

Suboptimal nutrition among children remains a problem among South Asian (SA) families. Appropriate complementary feeding (CF) practices can greatly reduce this risk. Thus, we undertook a systematic review of studies assessing CF (timing, dietary diversity, meal frequency and influencing factors) in children aged <2 years in Pakistan.

**Design:**

Searches between January 2000 and June 2016 in MEDLINE, EMBASE, Global Health, Web of Science, OVID Maternity & Infant Care, CINAHL, Cochrane Library, BanglaJOL, POPLINE and WHO Global Health Library. Eligibility criteria: primary research on CF practices in SA children aged 0–2 years and/or their families. Search terms: ‘children’, ‘feeding’ and ‘Asians’ with their derivatives. Two researchers undertook study selection, data extraction and quality appraisal (EPPI-Centre Weight of Evidence).

**Results:**

From 45 712 results, seventeen studies were included. Despite adopting the WHO Infant and Young Child Feeding guidelines, suboptimal CF was found in all studies. Nine of fifteen studies assessing timing recorded CF introduced between 6 and 9 months. Five of nine observed dietary diversity across four of seven food groups; and two of four, minimum meal frequency in over 50 % of participants. Influencing factors included lack of CF knowledge, low maternal education, socio-economic status and cultural beliefs.

**Conclusions:**

This is the first systematic review to evaluate CF practices in Pakistan. Campaigns to change health and nutrition behaviour are needed to meet the substantial unmet needs of these children.

Pakistan is one of the worst performers in child mortality in the world, with an under-5 mortality rate of 81 per 1000 live births in 2015^(^ ^1^ ^)^. Widespread malnutrition is reported in over 50 % of children living in Pakistan^(^ ^2^ ^)^. Both over- and undernutrition are prevalent within Pakistani families, with coexisting nutrition-related diseases; while 45 % of children have stunted growth, 40 % of Pakistani school-aged children are overweight^(^ ^3^ ^–^ ^7^ ^)^. Good feeding practices in early childhood should be a key area of focus to prevent malnutrition and measures to improve complementary feeding (CF) have been recommended for future investment^(^ ^8^ ^–^ ^10^ ^)^.

The WHO defines CF as: ‘The process starting when breast milk alone is no longer sufficient to meet the nutritional requirements of infants, and therefore other foods and liquids are needed, along with breast milk’^(^ ^11^ ^)^. CF therefore focuses on bridging the gradual transition between 6 and 24 months from exclusive breast-feeding to solid foods eaten by the whole family alongside breast-feeding. An appropriate diet following WHO guidelines in the first 2 years with breast-feeding and CF can decrease all-cause childhood mortality by approximately 20 % in low-income countries^(^ ^9^ ^)^. It can also guarantee sufficient nutritional intake, reducing obesogenic dietary behaviours while ensuring healthy gain of weight in children^(^ ^8^ ^)^.

The 2010 WHO Infant and Young Child Feeding (IYCF) guidelines, an internationally ratified framework adopted in Pakistan, emphasize as a global public health recommendation that infants should be exclusively breast-fed for the first 6 months of life. Thereafter, infants should receive safe and nutritionally adequate complementary foods while breast-feeding continues for up to 2 years of age or beyond^(^ ^12^ ^)^. The period from 6 months to 24 months of age is a critical and vulnerable period of infant growth^(^ ^13^ ^)^.

Current literature shows that knowledge of infant feeding practices and CF is lacking among Pakistani families, with Demographic and Health Survey (DHS) data from Pakistan highlighting inadequate breast-feeding and complementary feeding practices (CFP)^(^ ^14^ ^,^ ^15^ ^)^. The Pakistan DHS has only recently begun collecting information on all IYCF indicators, so an examination of these factors is particularly important^(^ ^16^ ^,^ ^17^ ^)^.

The present systematic review is part of a large project exploring the adequacy of CFP based on IYCF recommended minimum dietary diversity and meal frequency, timing of introducing CF, and barriers and promoters influencing CFP among South Asians (SA). SA communities have fundamental differences in geography and religion, which both influence feeding practices. As such, the current review is one of four looking at CFP in Pakistan, India, Bangladesh, and SA living in high-income countries. The work aims to inform future projects to develop and evaluate culturally appropriate interventions to improve CFP across SA families in these different communities.

## Methods

The present review (PROSPERO registration number CRD42014014025) summarizes publications on CFP in SA families in Pakistan only, with concurrent reviews summarizing publications on CFP in SA families in India^(^ ^18^ ^)^, Bangladesh^(^ ^19^ ^)^ and high-income countries (L Manikam, R Lingam, I Lever *et al*., unpublished results), respectively.

### Eligibility criteria

Studies were included if they met the following criteria.

∙ Participants: children aged 0–2 years, parents, carers and/or their families.
∙ Outcomes: adequacy of CF (based on minimum dietary diversity and meal frequency), timing of introduction of CF and barriers/promoters to incorporating WHO recommended CFP.
∙ Language: studies published in English, or with translation available.
∙ Year: published from 2000 or later.

In the IYCF indicators, introduction of CF is assessed as the proportion of infants aged 6–8 months who receive solid, semi-solid or soft foods^(^ ^20^ ^)^. In contrast, minimum dietary diversity (MDD) is assessed by the proportion of infants 6–23 months of age who receive foods from four or more food groups. The seven WHO IYCF recommended food groups are^(^ ^20^ ^)^:

1. grains, roots and tubers;
2. legumes and nuts;
3. dairy products (e.g. milk, yoghurt, cheese);
4. flesh foods (e.g. meat, fish, poultry and liver/organ meats);
5. eggs;
6. vitamin A-rich fruits and vegetables; and
7. other fruits and vegetables.

While the consumption of Fe-rich or Fe-fortified foods is commonly assessed as a separate IYCF indicator, this was incorporated within dietary diversity for ease of interpretation in the current review.

Finally, minimum meal frequency (MMF) is assessed by the proportion of breast-fed and non-breast-fed children 6–23 months of age who receive solid, semi-solid or soft foods (also including milk feeds for non-breast-fed children) the minimum number of times or more per day: two times for 6–8 months, three times for 9–23 months and four times for 6–23 months (if not breast-fed)^(^ ^20^ ^)^.

All study types (qualitative/quantitative/mixed) were included to ensure the diversity of evidence was captured and summarized, to be of relevance to both policy makers and health and social care professionals. We excluded studies focusing solely on exclusive breast-feeding and interventional studies.

### Information sources

We searched the following databases: MEDLINE, EMBASE, Global Health, BanglaJOL, CINAHL, Web of Science, OVID Maternity & Infant Care, The Cochrane Library, POPLINE and WHO Global Health Library. Searches were conducted in December 2014 and updated in June 2016.

Members of electronic networks on @jiscmail.ac.uk including minority-ethnic-health and networks (e.g. South Asian Health Foundation) developed from the Specialist Electronic Library for Ethnicity and Health were contacted to request any additional or unpublished material from members of the networks. Bibliographies of included articles were also hand-searched for additional publications.

### Search strategy

The search strategy included terms for ‘feeding’, ‘South Asian’ (including terms specifying all major subgroups as below) and ‘children’. For example, the search strings used for MEDLINE were the following.

Term 1: children <2 years

Infant OR Baby OR Babies OR Toddler OR Newborn OR Neonate* OR Child OR Preschool OR Nursery school OR Kid OR Paediatric* OR Minors OR Boy OR Girl

Term 2: feeding

Nutritional Physiological Phenomena OR Food OR Feeding behaviour OR Feed OR Nutrition OR Wean OR fortify* OR Milk

Term 3: Asians

Ethnic* OR India* OR Pakistan* OR Bangladesh* OR Sri Lanka OR Islam OR Hinduism OR Muslim OR Indian subcontinent OR South Asia

### Study selection and data extraction

In total, 45 712 titles and abstracts were screened against inclusion criteria. Two reviewers assessed these papers independently and conflicts were resolved by discussion with the team. In view of the large number of articles deemed eligible for full-text review, articles published before the year 2000 were excluded. In total, 44 852 titles and abstracts were excluded.

This left 860 articles describing CFP in SA children for full-text review by two independent reviewers. One hundred and thirty-one full-text articles were ultimately extracted, of which seventeen were sufficiently relevant to Pakistan.

Data were extracted by a single reviewer using a piloted modified worksheet including: country of study; study type; study year; study objectives; population studied, eligibility criteria and illness diagnosis; study design; ethical approval; sampling; data collection and analysis; feeding behaviours; adequacy of CFP; timing of initiation of CF; bias; value of the research; and weight of evidence. A second member of the research team checked each extraction.

### Result synthesis

The eligible studies tended to address broad research questions, were conducted using qualitative and/or quantitative and/or descriptive methods, and were not presented following standardized reporting guidelines (i.e. STROBE (Strengthening the Reporting of Observational Studies in Epidemiology) for observational studies or COREQ (Consolidated Criteria for Reporting Qualitative Research) for qualitative research). Meta-analyses were therefore not undertaken.

To standardize study classifications, the formal definitions below were applied.

1. Interventional study: a study in which patients are assigned to a treatment group or a comparison group and followed prospectively.
2. Cohort study: an observational study in which a group of patients are followed over time. These may be prospective or retrospective.
3. Cross-sectional study: an observational study that examines the relationship between health-related characteristics and other variables of interest in a defined population at one particular time.
4. Case–control study: a study that compares patients who have a disease or outcome of interest (cases) with patients who do not have the disease or outcome of interest (controls).
5. Qualitative: a study which aims to explore the experiences or opinions of families through interviews, focus groups, reflective field notes and other non-quantitative approaches.
6. Mixed methods: a study that combines both quantitative and qualitative methodology.

In view of the studies’ heterogeneity regarding methodology, participants, interventions and outcomes, a narrative approach to synthesis was utilized using guidance developed from the University of York Centre for Reviews and Dissemination (CRD) and the Economic and Social Research Council (ESRC)^(^ ^21^ ^–^ ^24^ ^)^.

The evidence reviewed is presented as a narrative report, with the results categorized following IYCF indicators on: (i) adequacy of CFP, comprising dietary diversity, consumption of Fe-rich foods, meal frequency, timing of introduction of CFP, food hygiene and sources of advice for feeding; and (ii) barriers/promoters influencing CFP.

Barriers were defined as obstacles or impediments to achieving correct CFP, while promoters were defined as supporters to achieving correct CFP. These were sub-categorized into factors influencing at the family level (e.g. family members) and the organizational level (e.g. health-care providers, hospitals, political bodies).

### Quality assurance

The CRD guidance emphasizes the importance of using a structured approach to quality assessment when assessing descriptive or qualitative studies for inclusion in reviews. However, it acknowledges the lack of consensus on the definition of poor quality with some arguing that using rigid quality criteria leads to the unnecessary exclusion of papers^(^ ^21^ ^)^.

In our review, the EPPI-Centre Weight of Evidence Framework was used to allow for objective judgements about the value of each study in answering the review question^(^ ^25^ ^)^. It examines three study aspects: quality of methodology, relevance of methodology and relevance of evidence to the review question, and categorizes them into ‘low’ (L), ‘medium’ (M) or ‘high’ (H). The overall weight of evidence (WOE), i.e. the overall assessment of the extent to which the study provides evidence to answer the review question, is also rated as L, M or H. Two independent reviewers performed this evaluation, with additional arbitration by other team members where required. One study with an overall WOE=L is included in the table summarizing included studies but is not discussed further within the ‘Results’ or ‘Discussion’ section below.

## Results

**Fig. 1:**
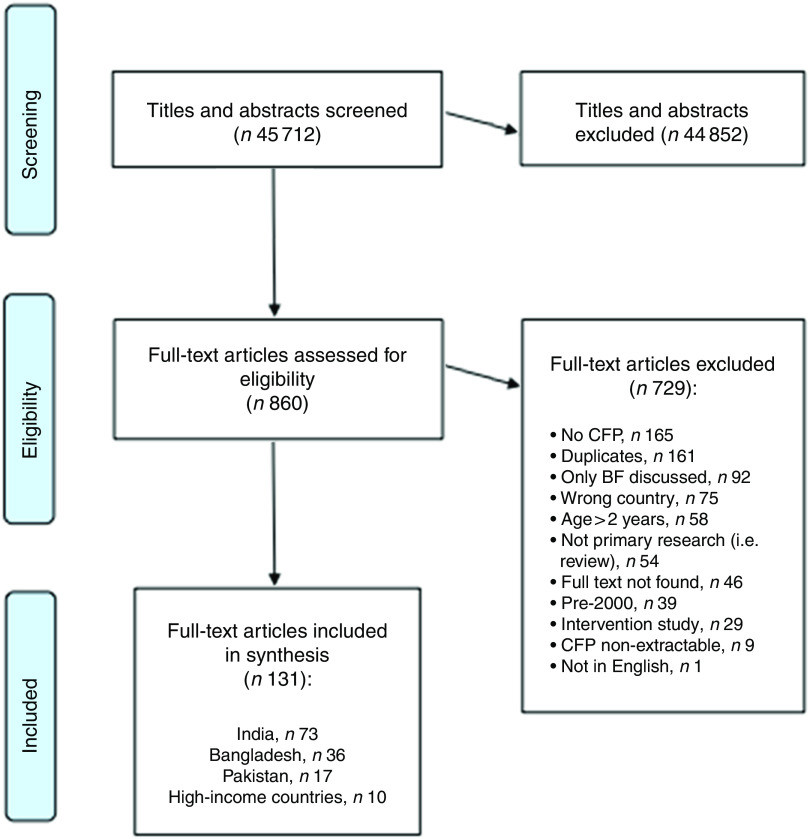
Study selection process for the current systematic review (CFP, complementary feeding practices; BF, breast-feeding)

Of the 45 712 studies identified, seventeen studies focusing on CFP in Pakistan were ultimately included in the current systematic review. The study selection process is denoted in Fig. 1.

### Study and participant characteristics

**Fig. 2:**
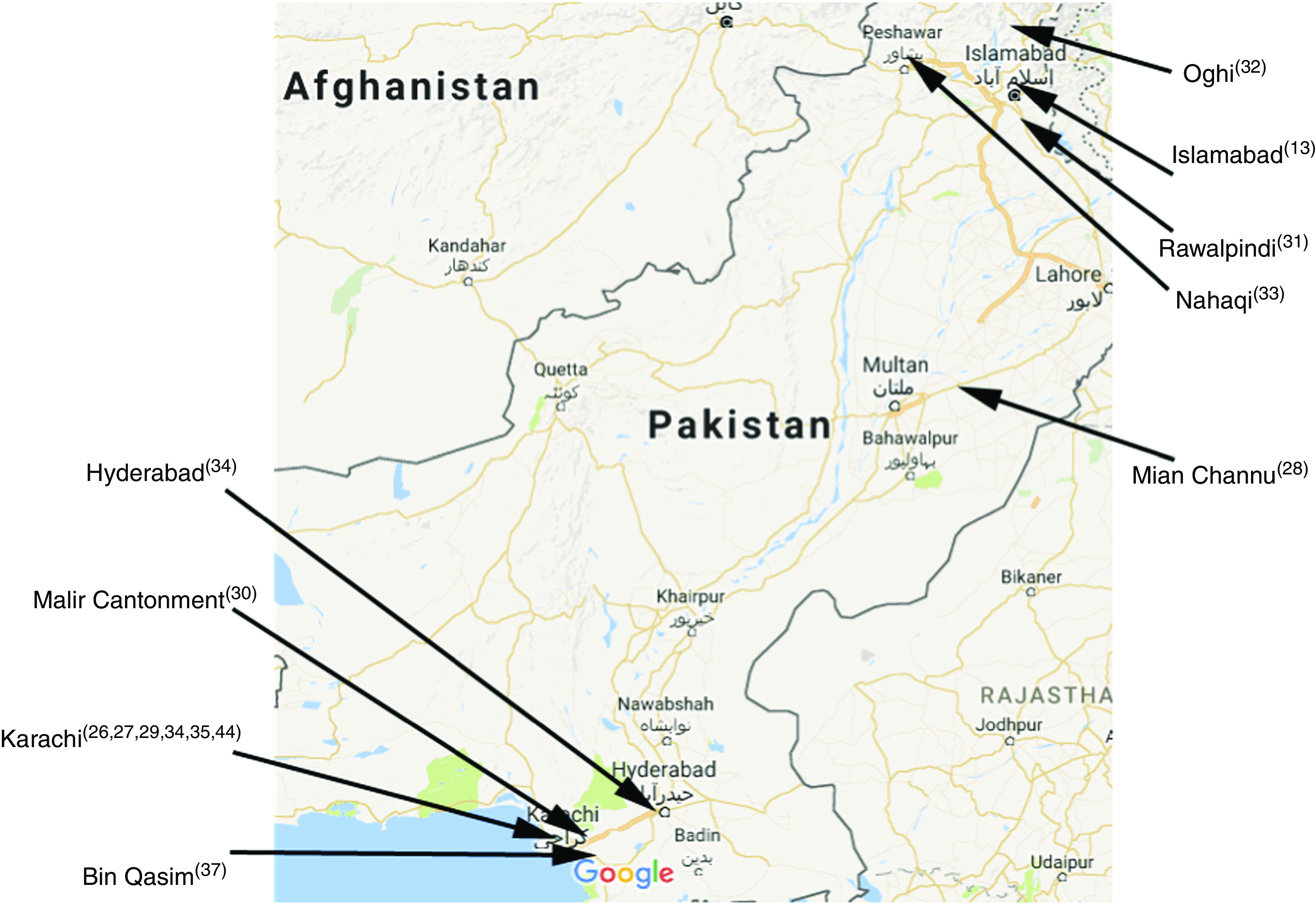
(colour online) Location map of fourteen of the studies included the current systematic review (map courtesy of Google Maps; data © 2017 Google)

These seventeen studies consisted of fourteen cross-sectional studies, two qualitative studies and one cohort study. Table 1 summarizes all included studies. Figure 2 illustrates the study locations of fourteen of these seventeen included studies, with the remaining three not detailing precise study locations.

**Table 1:** Summary of studies included in the current systematic review

Study	Study type	Location	Population	Sample size	Key findings
Ahmed *et al*. (2001)^(^ ^32^ ^)^	Cross-sectional	Oghi, Pakistan	Mothers of children attending the Tehsil Headquarter Hospital of Oghi	Ten mothers from test epicentre	Diversity: Milk delivered can include cow’s milk, formula, mixed milk. CF foods include banana, cereals, apple, home-made semi-solid foods Introduction to CF: 10 % with cereal, 30 % fruits, 10 % cow’s milk. For current feeds: 20 % with cereal, 30 % apples, 30 % bananas, 10 % cow’s milk Advice: Knowledge is transferred from one generation of women to the other in the household, advice also comes from women’s magazines, radio and television Factors: Lack of knowledge, especially where there are fewer babies, is a barrier
Dev *et al*. (2013)^(^ ^34^ ^)^	Cross-sectional	Karachi, Pakistan	Bilal Colony and Bhains Colony	355 mothers of children aged <2 years across two sites	Diversity: Bilal mothers used home food preparations more often than Bhains mothers. 93·0 % of home-made foods given in by sampled mothers in Bhains in Karachi contained *kitchri* (rice and pulses), as did 42·6 % of home-made foods given in Bilal. 21 % of mothers in Bilal provided only shop-bought/processed complementary foods *v*. 37 % in Bhains. Various combinations of mashed potato, banana, *khichri*, *sago dana*, *dalia* and others Frequency: In Bilal, 48·3 % fed 3+ times/d, Bhains 35·7 % fed 3+ times/d; and 10·2 and 9·5 % only 1 time/d Timing: In Bilal, 19·9 % before 6 months, 27·8 % at 6 months, 27·3 % at 7–11 months, 15·3 % at 1–2 years; in Bhains, 36·3, 5·0, 26·3 and 18·4 %, respectively (others ‘don’t know’) Advice: Cultural norms are spread within families, which may conflict with advice from health workers Factors: Difficulty with feeding was a barrier, also lack of knowledge. NGO activity increased knowledge
Dykes *et al*. (2012)^(^ ^33^ ^)^	Cohort	Nahaqi in Khyber Pakhtoonkhwa (KP), Pakistan	Women health workers in communities served by the Emergency Satellite Hospital in Nahaqi, KP	Sixteen local women health workers	Diversity: Weaning foods include *roti* (bread), rice, potato and banana Timing: Commencement of CF before 6 months is common, categorized 3–6 months Factors: Poverty stops women from affording e.g. milk, meat, fruits; affordability limits ability to provide ‘healthy’ foods; and insufficient milk causes early CF. If a mother becomes pregnant she is likely to cease breast-feeding or space feeds out
Hanif (2011)^(^ ^14^ ^)^	Cross-sectional	Pakistan	Evaluation survey using data from across Pakistan	168 332 households in Pakistan from 1990 to 2008	Timing: Introduction of CF foods was rated as ‘poor’ relative to WHO indicators; this was also the case for bottle-feeding and early initiation of breast-feeding. 32·1 and 36·3 % were having CF at 6–9 months in 1990–91 *v*. 2006–07
Hazir *et al*. (2012)^(^ ^15^ ^)^	Cross-sectional	Urban and rural Pakistan	Pakistan Demographic and Health Survey 2006–07 participants	941 infants	Timing: Over 50 % of infants were not receiving soft, semi-solid or solid foods at the recommended time; 10 % of 3–5-month-olds commenced CF earlier than the recommended 6 months. Early CF was related to 4+ antenatal visits and there were geographical variations. Urban respondents were more likely to engage in early and timely CF, with 13·8 % giving CF at 3–5 months and at 56·6 % at 6–8 months. Only 9·1 % of rural respondents gave CF at 3–5 months, and only 33·2 % at 6–8 months Factors: Literate mothers, those with high levels of education, with many antenatal visits, from urban households and from wealthy households were more likely to practise appropriate CF. Mothers in employment, with fewer antenatal visits, from rural households, and in the Baluchistan and Sindh regions were less likely to practise appropriate CF. Risk of not introducing CF at the recommended age was higher with increased parity. Generally, as wealth index increased, so too did appropriate CF timing
Krebs *et al*. (2011)^(^ ^26^ ^)^	Cross-sectional	Urban Karachi, Pakistan (together with Zambia, Guatemala and Democratic Republic of Congo)	Clinic visitors in Karachi	531 infants aged 5–9 months and 516 toddlers aged 12–24 months	Diversity: Meat is consumed often by under 25 % of infants; over 50 % receive fish, eggs or dairy regularly; 23·3 % are fed formula regularly. Legumes (beans and lentils), groundnuts (peanuts), meat, fish, eggs, dairy used Frequency: 50 % given 1–2 types of CF/d and 37·7 % receive CF 3+ times/d Factors: The cost of meat is a barrier, although modest amounts seem to be available in most households
Liaqat *et al*. (2007)^(^ ^13^ ^)^	Cross-sectional	Islamabad, Pakistan	Patients attending Outpatient Paediatrics Department of Federal Government Services Hospital, Islamabad	500 mothers of infants aged 6–24 months	Timing: CF starts as late as 12 months among 64 % of uneducated respondents, *v*. 17 % of educated respondents. Also, early at 3–6 months and 6–9 months optimal practice Advice: The extended family has an important role in supporting the mother and caring for the children Factors: Maternal education is positively linked to good CF practices, including appropriately timed initiation. Poverty is a key reason for inappropriate CF
Lingam *et al*. (2014)^(^ ^31^ ^)^	Qualitative	Rawalpindi district in Pakistan (and rural Udaipur district of Rajasthan in India)	Patients in Rawalpindi district	Sixty-nine contacts	Diversity: Diet is commonly based on grains and legumes. Cerelac, a store-bought porridge, was common among affluent families and used with boiled potato and banana. *Kitchri* is used for weaning with pulses and rice pudding Timing: Weaning started between 4 and 8 months Factors: Familial wealth was the main predictive factor for the age at introduction, interaction while feeding and dietary content of CF. Poorer households were less likely to have appropriate CF. Cultural beliefs had a large influence on CF. Parenting support important
Mehkari *et al*. (2014)^(^ ^27^ ^)^	Cross-sectional	Tertiary care hospital of Karachi, Pakistan	Female health-care professionals working at the hospital	Ninety-four mothers	Diversity: 85 % of mothers preferred home-made CF foods, 15 % used commercial preparations. Common CF food items included *dayla* (porridge, 64 %), *kitchri* (rice and pulses, 69 %), *kheer* (rice with milk, 48 %) and mashed potato (65 %) Timing: 78 % of mothers knew CF should be initiated at 6 months, 53·2 % followed this up in practice. Weaning also initiated at 5 and 4 months Factors: Professional and employment-related issues resulted in early cessation of breast-feeding
Memon *et al*. (2010)^(^ ^36^ ^)^	Cross-sectional	Liaquat University Hospital Jamshoro, Hyderabad, Pakistan	Mothers of infants aged 0–24 months	500 mothers	Diversity: Of CF children aged 6–11 months (with 24 h recall), 40 (33 %) were given specially prepared meals Frequency: 100 (50 %) children aged 12–23 months received CF at appropriate frequency of 3–4 times/d Timing: 21 % of 2–3-month-olds received CF. 50 % of 12–23-month-olds received 3–4 CF feeds/d, categorized <3, 3–6 months Advice: For 78 % CF advice was given by family, for 22 % by doctors and health workers Factors: CF practices correlated with maternal education. Poorer households were more likely to have inadequate feeding practices than well-to-do families. Some mothers received incorrect advice from health workers. Mother getting pregnant resulted in failure to breast-feed for full 6 months. Being poor rather than middle class had an impact. Some initiated formula as breast milk was insufficient
Mohsin *et al*. (2014)^(^ ^35^ ^)^	Cross-sectional	Outpatient Department of Pediatrics, Civil Hospital, Karachi, Pakistan	Mothers attending Outpatient Department of Pediatrics, Civil Hospital, Karachi	138 mothers of children aged up to 2 years	Diversity: Quality and quantity of CF often inadequate. 46·4 % used commercial food, tea used for CF Frequency: CF frequency often inadequate; 55·8 % fed solid foods 3 times/d, 39·9 % twice/d, 2·2 % once/d Timing: 84 % had knowledge that optimal time for CF was <6 months, only 13 % knew about correct timing. CF was often started early. 27·5 % considered <4 months to be optimal, and 57·2 % 4–6 months Advice: Doctors were main source of information about commercial CF foods for 52·9 %, *v*. friends and relatives for 25·4 % and electronic media for 17·4 % Factors: Cultural beliefs about certain hot or cold foods; mothers unaware of appropriate times for CF; lack of maternal time
Premji *et al*. (2014)^(^ ^37^ ^)^	Qualitative	Ibrahim Haidery, Bin Qasim town of Karachi, Pakistan	Families with infants less than 6 weeks old	Ten mothers, eight fathers and four grandmothers	Timing: CF observed at <3 months, such as of *sulemnai chai*, and supplementing after 40 d Advice: Extended family members are very important for support in resolving issues of feeding and childcare. Elders outside the family were also consulted Factors: Cultural influences played a big part in CF, particularly in introducing non-breast-milk foods into diets before 6 months. Cultural beliefs can especially be spread by elders
Sarwar (2002)^(^ ^28^ ^)^	Cross-sectional across two countries	Mian Channu, Pakistan and England, UK	Pakistani mothers in England and Pakistan with children aged 3–12 months	Ninety mothers (forty-five living in England and forty-five in Pakistan)	Diversity: Family foods were used for CF in Pakistan, *v.* vegetables, sweet convenience foods and meat in England. In Pakistan, first introduced to CF with: 42 % cereal, 49 % rice, 11 % fruits, 11 % vegetables, 20 % egg, 2 % sweet convenience foods, 2 % meat. At time of study: 84 % rice, 29 % cereal, 60 % fruits, 47 % vegetables, 44 % egg, 11 % savoury convenience foods, 20 % sweet convenience foods, 24 % meat. Tea was also used for CF Timing: 40 % in Pakistan introduced solids at 3–4 months, 26 % did not wean until 7 months or later Advice: Most mothers, two-thirds, received advice on weaning from family and friends. Health professionals also advised Factors: Delayed CF occurred due to beliefs breast milk was nutritionally adequate. Facilitating factors included booklets in English and Urdu, audio tapes in traditional language, group discussions and clinic demonstrations, and integrating health education into primary care
Senarath *et al*. (2012)^(^ ^16^ ^)^	Cross-sectional	Pakistan	Mothers aged 12–49 years of from most recent Demographic and Health Survey data of Pakistan (2006–07)	443 infants aged 6–8 months	Timing: Introduction of solid, semi-solid, or soft foods occurred for 39 % of Pakistan infants aged 6–8 months – low relative to other countries Factors: Delayed CF was significantly more likely in poorer households relative to the richest; birth order 5+ also a barrier
Shamim (2005)^(^ ^29^ ^)^	Cross-sectional	Peri-urban Karachi, Pakistan	Factory workers from multiple ethnic groups visiting ‘Well Baby Clinic’ in Jinnah Medical College Hospital	150 infants	Diversity: Tea is used extensively in >50 % of children. *Kitchri*, *dayla* and *suji* were also used for CF. Banana only fruit offered for CF. Egg seldom used for CF. Frequent use of milk-based cereal. 52 % of mothers used commercial cereals. Meat almost never used Timing: Mothers with worse education sometimes delayed CF until the infant was 1 year old, general range within participants was 3–9 months Advice: Family members and friends (32 %), media (23 %), medical/paramedical staff (6·7 %) Factors: Delayed CF was associated with low educational status of mothers, and with larger family sizes and time pressure on mothers. Late weaning occurred in 34·2 % of those with parity <3 and 59·6 % of those with parity 3+. Food was diluted to save money. Custom/not enough breast milk also a factor
Shamim *et al*. (2006)^(^ ^30^ ^)^	Cross sectional	Rural areas of Malir district in Karachi, Pakistan	Families living in Malir district	359 children under 3 years old	Diversity: Two types of CF items observed – home-made food (home-made cereals, egg, banana, fish) and commercial food (read-to-use cereals, biscuits and rusk). Even those who ate the former group had high prevalence of malnourishment, stunting and wasting. *Kitchri*, *dalia*, *suji* used for weaning. Fish is given regularly in fishing villages but is too costly elsewhere Timing: 296 were weaned between 4 and 6 months, sixty-three outside this range Factors: Food affordability and access – banana is given due to its easy access, fish less often due to expense
Sultana *et al*. (2011)^(^ ^43^ ^)^	Cross-sectional	Karachi, Pakistan	Vaccination centre in the Outpatient Department of Paediatrics, Civil Hospital	200 children aged 4–6 months	Timing: Direct relationship between education status of mother and children being breast-fed for 4 months Factors: Illiterate mothers or less educated mothers had inappropriately timed CF

CF, complementary feeding/complementary food; NGO, non-governmental organization.

Table 2 presents the weight of evidence awarded to each of the studies. The core narrative themes extracted from the papers are presented under the following headings: (i) adequacy of CFP and (ii) factors associated with CFP.

**Table 2:** Weight of evidence awarded to each study in the current systematic review

	Weight of Evidence A	Weight of Evidence B	Weight of Evidence C	Weight of Evidence D
Study	Quality of methodology (accuracy, coherency and transparency of the evidence)	Relevance of methodology (appropriateness of the methodology for answering the review question)	Relevance of evidence to the review question (relevance of the focus of the evidence for answering the review question)	Overall weight of evidence (overall assessment of the extent to which the study provides evidence to answer the review question)
Ahmed *et al*.^(^ ^32^ ^)^	L	M	M	M
Dev *et al*.^(^ ^34^ ^)^	H	H	H	H
Dykes *et al*.^(^ ^33^ ^)^	L	M	M	M
Hanif^(^ ^14^ ^)^	M	M	M	M
Hazir *et al*.^(^ ^15^ ^)^	H	M	H	H
Krebs *et al*.^(^ ^26^ ^)^	M	M	M	M
Liaqat *et al*.^(^ ^13^ ^)^	M	M	M	M
Lingam *et al*.^(^ ^31^ ^)^	M	H	H	H
Mehkari *et al*.^(^ ^27^ ^)^	H	M	H	H
Memon *et al*.^(^ ^36^ ^)^	M	H	H	H
Mohsin *et al*.^(^ ^35^ ^)^	M	H	H	H
Premji *et al*.^(^ ^37^ ^)^	M	M	L	M
Sarwar^(^ ^28^ ^)^	H	H	H	H
Senarath *et al*.^(^ ^16^ ^)^	H	H	M	H
Shamim^(^ ^29^ ^)^	M	M	H	M
Shamim *et al*.^(^ ^30^ ^)^	H	M	M	M
Sultana *et al*.^(^ ^44^ ^)^	L	M	L	L

L, low; M, medium; H, high.

### Adequacy of complementary feeding practices

As per the WHO IYCF indicators, adequacy of CFP is assessed according to dietary diversity, meal frequency and timing of introducing CFP. These are detailed in the subsections below, with three further subsections discussing Fe-rich foods, food hygiene and advice providers.

#### Dietary diversity

Dietary diversity was explored in nine studies, with food consumption recall being the commonest way to assess MDD. Most studies did not provide precise rates of the population being fed with each food group, and no study properly assessed rates of achieved MDD across four of the seven WHO IYCF food groups defined above.

Table 3 denotes a summary of all complementary food groups identified from the studies. Of the nine studies with data relevant to MDD, five studies described CFP involving four of the seven food groups^(^ ^26^ ^–^ ^30^ ^)^. Overall, Pakistani CF diets were described as being based largely on grains and legumes by Lingam *et al*. (WOE=H)^(^ ^31^ ^)^. Shamim *et al*. (WOE=M) observed frequent poor dietary diversity, with even those who were fed recommended weaning items (including home-made cereals, egg, banana and fish) having a high prevalence of stunting (31·4 %), wasting (20·3 %) and underweight (46·4 %)^(^ ^30^ ^)^.

**Table 3:** Foods utilized for complementary feeding in Pakistan, categorized into WHO food groups

WHO classified food group	Number of studies and references
Grains, roots and tubers	Nine studies^(^ ^26^ ^–^ ^34^ ^)^
Legumes and nuts	Six studies^(^ ^26^ ^,^ ^27^ ^,^ ^29^ ^–^ ^31^ ^,^ ^34^ ^)^
Dairy products (e.g. milk, cheese, yoghurt)	Five studies^(^ ^26^ ^–^ ^28^ ^,^ ^32^ ^)^
Flesh foods (e.g. meat, fish, poultry, and liver/organ meats)	Four studies^(^ ^26^ ^,^ ^28^ ^–^ ^30^ ^)^
Eggs	Four studies^(^ ^26^ ^,^ ^28^ ^–^ ^30^ ^)^
Vitamin A-rich fruits and vegetables	Zero studies
Other fruits and vegetables	Eight studies^(^ ^27^ ^–^ ^34^ ^)^

If type of fruit/vegetable was not provided, it was classified as ‘other fruits and vegetables’.

In nine studies, the ‘grains, roots and tubers’ food group was utilized for CFP. Eight named ‘other fruits and vegetables’, six mentioned ‘legumes and nuts’, five studies included ‘dairy products’, four studies ‘flesh foods’ and four studies ‘eggs’. No studies mentioned ‘vitamin A-rich fruits and vegetables’.

‘Grains, roots and tubers’ were most commonly mentioned as being used for CF, included in nine studies. Sarwar (WOE=H) observed that 42 % of sampled children aged 3–12 months were first introduced to CF with cereal, and 49 % with rice; at the time of the study, 84 % were fed rice and 29 % cereal^(^ ^28^ ^)^. In comparison, Ahmed *et al*. (WOE=M) found that 10 % were introduced to CF with cereal, and 20 % currently were fed rice^(^ ^32^ ^)^. Mehkari *et al*. (WOE=H) listed common CF food items including *daliya* (porridge, 64 %), several made with rice such as *kitchri* (rice and pulses, 69 %) and *kheer* (rice with milk, 48 %), and mashed potato (65 %)^(^ ^27^ ^)^. Shamim (WOE=M) also listed *kitchri*, *daliya*/*dayla* and *suji* (a wheat derivative) as CF foods^(^ ^29^ ^)^. Lingam *et al*. (WOE=H) observed that Cerelac, a store-bought porridge, was common among affluent families, and in some families, it was mixed with boiled potatoes^(^ ^31^ ^)^.

‘Other fruits and vegetables’ were mentioned in eight studies with none directly specifying use of ‘vitamin A-rich fruits and vegetables’. Sarwar (WOE=H) observed that 11 % of infants aged 3–12 months were first introduced to CF with fruits and 11 % with vegetables, and 60 % were currently fed fruits and 47 % vegetables^(^ ^28^ ^)^; while Ahmed *et al*. (WOE=M) noted that 30 % were first weaned with fruits of any sort, while 30 % were currently fed apples and 30 % bananas^(^ ^32^ ^)^. Banana was popular, with Shamim (WOE=M) finding that banana was the only fruit offered by participants, and that seasonal fresh fruit and vegetables were not offered at all^(^ ^29^ ^)^. Shamim *et al*. (WOE=M) and Dykes *et al*. (WOE=M) also observed the use of banana^(^ ^30^ ^,^ ^33^ ^)^.

‘Legumes and nuts’ were identified in six studies for CF, primarily through the use of pulses in *kitchri*, a dish made of pulses with rice. Dev *et al*. (WOE=H) found that 93·0 % of home-made food given by sampled mothers in Bhains Colony in Karachi contained *kitchri*, as did 42·6 % of home-made food given in Bilal Colony^(^ ^34^ ^)^; and Mehkari *et al*. (WOE=H) found that 69·1 % of participants used *kitchri* for CF^(^ ^27^ ^)^. Shamim *et al*. (WOE=M), Shamim (WOE=M) and Lingam *et al*. (WOE=H) also listed *kitchri* as a weaning food^(^ ^29^ ^–^ ^31^ ^)^. Krebs *et al*. (WOE=M) described the use of beans and lentils, and groundnuts (peanuts)^(^ ^26^ ^)^.

In terms of ‘dairy products’, Sarwar (WOE=H) described the use of cow’s milk by 37 % of those sampled and Ahmed *et al*. (WOE=M) described its use by 10 %^(^ ^28^ ^,^ ^32^ ^)^. Shamim (WOE=M) described extensive use of milk-based cereals^(^ ^29^ ^)^. Sarwar also found that 20 % of infants were introduced to CF with eggs, and 44 % were currently fed eggs^(^ ^27^ ^)^, although conversely Shamim (WOE=M) claimed egg seldom comprises CF foods^(^ ^29^ ^)^.

The use of commercial foods was mentioned by six studies, with usage rates ranging from 11 to 52 %. Shamim (WOE=M) found that 52 % of mothers used commercial cereals for CF^(^ ^29^ ^)^, while Mohsin *et al*. (WOE=H) reported that 46·4 % used commercial food items in general^(^ ^35^ ^)^, and in Mehkari *et al*. (WOE=H) this proportion was 15 %^(^ ^27^ ^)^. Sarwar (WOE=H) claimed that 2 % of infants were introduced to CF with sweet convenience foods, and at the time of the study 11 % were regularly consuming savoury convenience foods and 20 % sweet convenience foods^(^ ^28^ ^)^. Dev *et al*. (WOE=H) reported that 21 % of mothers in Bilal Colony provided only shop-bought/processed CF compared with 37 % in Bhains Colony, attributing this difference to the presence of a non-governmental organization in Bilal^(^ ^34^ ^)^. Shamim *et al*. (WOE=M) also mentioned the use of commercial foods^(^ ^30^ ^)^. Sarwar and Mohsin *et al*. reported tea was used for CF, which is not part of any WHO recommended food group^(^ ^28^ ^,^ ^35^ ^)^.

#### Iron-rich foods

There was little information on consumption of Fe-rich foods. Use of flesh foods (meat, fish, poultry and liver/organ meats) was mentioned in only four studies. In Sarwar (WOE=H), 2 % of infants were first introduced to CF with meat, and at the time of the study 24 % were fed with meat^(^ ^28^ ^)^. This is a similar proportion to Krebs *et al*. (WOE=M), who found that 22·2 % were fed meat regularly^(^ ^25^ ^)^. Conversely, Shamim (WOE=M) claimed that meat was almost never used for CF^(^ ^29^ ^)^. Otherwise, Shamim *et al*. (WOE=M) described fish as being given for CF ‘regularly’ in fishing villages, although it is considered too costly elsewhere^(^ ^30^ ^)^.

#### Meal frequency

Meal frequency was measured in four studies, with rates of MMF ranging from 35·7 to 55·8 % across the studies. MMF is two times for 6–8 months, three times for 9–23 months and four times for 6–23 months (if not breast-fed)^(^ ^14^ ^)^.

Mohsin *et al*. (WOE=H) found that 55·8 % of infants aged 0–2 years were fed solid foods three times daily, achieving MMF if paired with breast-feeding; they also found that 39·9 % were fed twice daily (which would meet MMF only for 6–8-month-olds) and 2·2 % were fed only once daily^(^ ^35^ ^)^. Memon *et al*. (WOE=H) found 50 % of infants aged 12–23 months received complementary foods at the MMF optimal rate of three or four times daily^(^ ^36^ ^)^. Krebs *et al*. (WOE=M) found that 37·7 % of infants aged 5–9 months were fed three or more times daily, meaning at least this proportion at a minimum met MMF, and 50·7 % were fed once or twice daily^(^ ^25^ ^)^. Comparing two colonies in Karachi, Dev *et al*. (WOE=H) found that 48·3 % were fed three or more times daily in Bilal Colony, but 35·7 % in Bhains Colony; as many as 10·2 and 9·5 % in the respective colonies were fed only once daily^(^ ^34^ ^)^.

#### Timing of introducing complementary feeding

Table 4 denotes a summary of timing of when CF was introduced across the studies. The most commonly listed age for CF commencement was at 3–6 months (eleven studies), followed by 6–9 months of age, the optimal period (nine studies). The third most common were under 3 months of age and 9–12 months of age, both by three studies. One study described CF introduction between 1 and 2 years.

**Table 4:** Timing of introduction of complementary feeding in Pakistan

Infant age	Number of studies and references
<3 months	Three studies^(^ ^35^ ^–^ ^37^ ^)^
3–6 months	Eleven studies^(^ ^13^ ^,^ ^15^ ^,^ ^27^ ^–^ ^31^ ^,^ ^33^ ^–36)^
6–9 months	Nine studies^(^ ^13^ ^–^ ^16^ ^,^ ^27^ ^–^ ^29^ ^,^ ^31^ ^,^ ^34^ ^)^
9–12 months	Three studies^(^ ^13^ ^,^ ^29^ ^,^ ^34^ ^)^
12–15 months	One study^(^ ^34^ ^)^
15–18 months	One study^(^ ^34^ ^)^
>18 months	Zero studies

Based on the 2006–07 Pakistan DHS, Senarath *et al*. (WOE=H) noted that timely initiation of CF in Pakistan was the lowest (39 %) compared with other SA countries: India achieved 55 %, Nepal 70 %, Bangladesh 71 % and Sri Lanka 84 %^(^ ^16^ ^)^. There was little improvement in the timing of introducing CF in infants aged 6–9 months between 1990–91 (32·1 %) and 2006–07 (36·3 %) in the national DHS, and the timing of introducing CF was ranked as ‘poor’ by Hanif (WOE=M)^(^ ^14^ ^)^; Hazir *et al*. (WOE=H) found 60·8 % of Pakistani infants aged 6–8 months had not commenced CF, with 10·6 % of infants starting CF earlier than the recommended time^(^ ^15^ ^)^.

Hazir *et al*. (WOE=H) found that urban respondents were more likely to practise early or timely CF, with 13·8 % giving CF at 3–5 months and at 56·6 % at 6–8 months^(^ ^15^ ^)^. Only 9·1 % of rural respondents gave CF at 3–5 months and only 33·2 % at 6–8 months^(^ ^15^ ^)^. Liaqat *et al*. (WOE=M) observed CF starting as late as up to 12 months among 64 % of uneducated respondents, compared with 17 % of educated respondents^(^ ^13^ ^)^, and Shamim (WOE=M) found that less educated mothers sometimes delayed CF until the infant was 1 year old^(^ ^29^ ^)^. Dev *et al*. (WOE=H) described CF beginning between 1 and 2 years for 15·3 % in Bilal Colony and for 18·4 % in Bhains Colony^(^ ^34^ ^)^.

#### Food hygiene

Two studies looked at hand-washing and found good compliance; Mohsin *et al*. (WOE=H) found that 92 % of respondents washed hands before cooking^(^ ^35^ ^)^, which is similar to Mehkari *et al*. (WOE=H) where 98 % practised hand-washing before feeding children^(^ ^27^ ^)^. However, Mohsin *et al*. also found that 71·7 % did not boil drinking-water^(^ ^34^ ^)^.

Meal preparation for infants was generally done together with other family cooking. Three studies described meal preparation, with Mohsin *et al*. (WOE=H) noting that 50 % of the mothers were cooking complementary foods separately from adult foods and Memon *et al*. (WOE=H) describing that only 33 % of children aged 6–11 months were provided with meals especially prepared for them^(^ ^35^ ^,^ ^36^ ^)^. Only 25·6 % of mothers who home-cooked food prepared meals especially for the infant in Shamim (WOE=M)^(^ ^29^ ^)^.

#### Advice providers

Nine studies discussed advice providers. Of these, eight studies highlighted the importance of advice from family and friends, and one indicated that advice from medical professionals predominated. Memon *et al*. (WOE=H) found that 78 % of mothers in their sample were advised primarily by family members, compared with 22 % by medical professionals^(^ ^36^ ^)^, and Sarwar (WOE=H) found that two-thirds of mothers received advice from family and friends on weaning^(^ ^28^ ^)^. Six other studies similarly emphasized the importance of families and friends in giving advice for CF^(^ ^13^ ^,^ ^29^ ^,^ ^32^ ^–^ ^34^ ^,^ ^37^ ^)^. Mohsin *et al*. (WOE=H) was the one study that indicated otherwise, saying that doctors were the primary source of knowledge in the case of commercial complementary foods for 52·9 %, compared with 25·4 % who consulted relatives and friends^(^ ^35^ ^)^. Three studies mentioned the media as another source^(^ ^29^ ^,^ ^32^ ^,^ ^35^ ^)^. Other sources included women’s magazines in Ahmed *et al*. and extra-familial elders in Premji *et al*.^(^ ^32^ ^,^ ^37^ ^)^.

### Factors associated with complementary feeding practices

We identified several factors influencing CFP. They are summarized in Table 5 as either a barrier or a promoter, and sub-categorized as acting at family or organizational level. Overall, eight promoters and fourteen barriers were identified.

**Table 5:** Factors influencing complementary feeding (CF) practices in Pakistan

Family level			
Promoters	Number of studies and references	Barriers	Number of studies and references
More educated mothers	Four studies^(^ ^13^ ^,^ ^15^ ^,^ ^29^ ^,^ ^36^ ^)^	Lack of maternal knowledge of CF	Five studies^(^ ^28^ ^,^ ^32^ ^,^ ^34^ ^–^ ^36^ ^)^
Literate mothers	One study^(^ ^15^ ^)^	Cultural beliefs	Four studies^(^ ^29^ ^,^ ^31^ ^,^ ^35^ ^,^ ^37^ ^)^
Mother who had 4+ antenatal visits	One study^(^ ^15^ ^)^	Higher parity of mother	Three studies^(^ ^15^ ^,^ ^16^ ^,^ ^29^ ^)^
Having a high level of parenting support	One study^(^ ^31^ ^)^	Insufficient breast milk	Three studies^(^ ^29^ ^,^ ^33^ ^,^ ^36^ ^)^
		Lack of maternal time	Two studies^(^ ^29^ ^,^ ^35^ ^)^
		Mother in employment	Two studies^(^ ^15^ ^,^ ^27^ ^)^
		Mother becomes pregnant during CF period	Two studies^(^ ^33^ ^,^ ^36^ ^)^
		Mothers who had <4 antenatal visits	One study^(^ ^15^ ^)^
		Difficulty getting the baby to feed	One study^(^ ^33^ ^)^
		Delivery by caesarean section	One study^(^ ^15^ ^)^
Organizational level			
Promoters	Number of studies and references	Barriers	Number of studies and references
Wealthy households	Three studies^(^ ^15^ ^,^ ^16^ ^,^ ^31^ ^)^	Household poverty	Six studies^(^ ^13^ ^,^ ^15^ ^,^ ^16^ ^,^ ^31^ ^,^ ^32^ ^,^ ^37^ ^)^
NGO activity	One study^(^ ^34^ ^)^	Food affordability and access	Four studies^(^ ^26^ ^,^ ^29^ ^,^ ^30^ ^,^ ^33^ ^)^
Improved health education	One study^(^ ^28^ ^)^	Poor advice from health workers	One study^(^ ^36^ ^)^
Urban households	One study^(^ ^15^ ^)^	Rural households	One study^(^ ^15^ ^)^

NGO, non-governmental organization.

#### Promoters

Five studies identified four promoters at the family level. Promoters were: more educated mothers (four studies); literate mothers (one study); mother who had more than four antenatal visits (one study); and high level of parenting support (one study).

The most commonly mentioned promoter was ‘more educated mothers’, identified in four studies^(^ ^13^ ^,^ ^15^ ^,^ ^29^ ^,^ ^36^ ^)^. A positive relationship between maternal education and nutritional status was described by Liaqat *et al*. (WOE=M)^(^ ^13^ ^)^, with Shamim (WOE=M) noting that some uneducated mothers delayed CF until their child was 1 year old^(^ ^29^ ^)^. Interestingly, while Memon (WOE=H) *et al*. noted that the probability of inappropriate CFP in uneducated mothers was 3·7 times higher than in educated mothers, there was no correlation between paternal education and CFP^(^ ^36^ ^)^.

Five studies identified four promoters at the organizational level. Promoters were: wealthy households (three studies); activity of non-governmental organization (one study); improved health education (one study); and urban households (one study).

The most identified promoter was wealthier households. Hazir *et al*. (WOE=H) found that the percentage of timely CF at 6–8 months was higher in richer and richest households, at 51·3 and 70·9 %, respectively, compared with poorest, poorer and middle households, which achieved rates of 28·0, 33·7 and 23·1 %, respectively^(^ ^15^ ^)^. This association was also found in Senarath *et al*. (WOE=H) and Lingam *et al*. (WOE=H)^(^ ^16^ ^,^ ^31^ ^)^.

#### Barriers

Twelve studies identified ten barriers at the family level. Barriers were: lack of maternal knowledge on CF (five studies); cultural beliefs (four studies); higher parity of mother (three studies); insufficient breast milk (three studies); lack of maternal time (two studies); mother in employment (two studies); mother becomes pregnant during CF period (two studies); mother who had fewer than four antenatal visits (one study); delivery by caesarean section (one study); and difficulty getting the baby to feed (one study).

The most cited family-level barriers were lack of maternal knowledge on CF and cultural beliefs. In terms of inadequate knowledge, Memon *et al*. (WOE=H) found that 90 % of mothers had inadequate knowledge on frequency of CF^(^ ^36^ ^)^, with large gaps in knowledge also reported by Mohsin *et al*. (WOE=H)^(^ ^35^ ^)^. Knowledge is not sufficient for good CF, however; Mehkari *et al*. (WOE=H) reported that although 78 % of female health-care professionals knew WHO guidelines on initiating CF at 6 months, only 53 % adhered to them^(^ ^27^ ^)^. Regarding cultural beliefs, ideas about certain foods being ‘hot’ or ‘cold’ inhibited dietary diversity. Mohsin *et al*. (WOE=H) listed ‘hot’ foods including bread, meat, potato and egg, and ‘cold’ foods including rice, curd and banana, and observed that 41 % of caregivers would avoid ‘cold’ foods in winter and during illness^(^ ^35^ ^)^. Premji *et al*. (WOE=M) also found that cultural beliefs inhibit CF during illness^(^ ^37^ ^)^.

In total, nine studies identified four barriers at the organizational level. Barriers were: household poverty (six studies); food affordability and access (four studies); poor advice from health workers (one study); and rural households (one study).

The barrier posed by being poor was the most popular influencing factor either positively or negatively. Hazir *et al*. (WOE=H) and Senarath (WOE=H) both identified that CF initiation was delayed significantly in the middle to poorest wealth index compared with the richest in Pakistan^(^ ^15^ ^,^ ^16^ ^)^. Dykes *et al*. (WOE=M) described how women felt that poverty stopped them being able to adequately feed their children^(^ ^33^ ^)^, and Liaqat *et al*. (WOE=M) named poverty as one of the main reasons for inappropriate practices^(^ ^13^ ^)^. Cost and accessibility was the second most identified barrier, with Shamim (WOE=M) finding that meat and seasonal fruits were rarely given due to their cost and low availability, and Shamim *et al*. (WOE=M) observing that expensive ready-to-make cereals were often overdiluted^(^ ^29^ ^,^ ^30^ ^)^.

## Discussion

To our knowledge, the present is the first systematic review to assess CFP in Pakistan. We identified that CFP in many Pakistani SA families were not meeting WHO IYCF standards on minimum dietary diversity, meal frequency and timing of introducing CF.

### Implications of key findings

Grains, roots, tubers and legumes appear to predominate for CF in Pakistan. It is concerning that only five of nine studies discussing dietary diversity described CF foods covering four of the seven groups recommended in the WHO IYCF guidelines; the true number of infants consuming foods across these four groups is likely to be low. No study directly addressed rates of meeting MDD requirements. Inappropriate CF foods were sometimes described, such as tea and crackers mentioned by Sarwar (WOE=H) and Shamim *et al*. (WOE=M)^(^ ^28^ ^,^ ^30^ ^)^. Besides reducing appetite, tea tannin impairs Fe absorption which may contribute to anaemia^(^ ^38^ ^)^. The widespread use of commercial complementary food items in Pakistan due to doctors’ recommendation, perceptions that this is ‘best for the baby’, media influence and easy availability of such foods is another issue which must be tackled^(^ ^35^ ^)^. Only two studies provided information on CF related hygiene, which both discussed hand-washing^(^ ^27^ ^,^ ^35^ ^)^. Food hygiene is an emerging concern in Pakistan and we recommend future studies collect data on this important topic^(^ ^39^ ^)^.

In terms of MMF, even when CF is initiated it is often not given at the optimum rate of three or four times daily. Increased presence of non-governmental organizations and medical staff in local factories may help improve CF practices; Dev *et al*. (WOE=H) examined CF in two socio-economically similar colonies in Karachi and linked the superior CF practices in Bilal Colony, where 48·3 % met MMF compared with 35·7 % in Bhains Colony, to these factors^(^ ^34^ ^)^. This approach could help inform interventions to improve MMF in other areas.

Timing of CF is a problem in Pakistan, which has the lowest proportion of timely CF among all the SA countries^(^ ^16^ ^)^. Premature CF is associated with increased risk of infection according to Shamim *et al*. (WOE=M)^(^ ^30^ ^)^, and Sarwar (WOE=H) found late introduction of CF affected growth and could lead to defiant eating behaviours including rejection of food and difficulty in learning to masticate^(^ ^28^ ^)^. Measures to increase knowledge about timing of CF can help mitigate these health effects.

The eight promoters identified in the present review can help inform interventions to improve CF in Pakistan. One in particular, improved health education, could help address some familial barriers such as a lack of knowledge or cultural beliefs. Sarwar (WOE=H) found that mothers wanted information in traditional languages to help improve their care practices, and also valued group discussions and demonstrations^(^ ^28^ ^)^. It was thought that such sessions, by being more interactive, could address points of confusion. Another health-care intervention, cognitive behavioural therapy, has been trialled to target deeply embedded beliefs around breast-feeding in a socio-economically disadvantaged district of Pakistan^(^ ^40^ ^)^. By building a therapeutic relationship with the mother, this holistic intervention was used successfully to deliver messages on breast-feeding. Active methods to encourage mothers to practise correct CF behaviour could be very effective, and should be practical while respecting the local customs and traditional culture^(^ ^28^ ^)^.

Fourteen separate barriers to appropriate CF were identified. Among the familial barriers, the listing of ‘mother in employment’ as a barrier is concerning and suggests that more needs to be done to support working mothers. Memon *et al*. (WOE=H) suggest workplace policies should be modified to provide social support and motivation for breast-feeding among working mothers^(^ ^36^ ^)^. A further very notable barrier is the absence of knowledge on appropriate CF at all levels. Previous studies have shown that educational and behaviour change interventions successfully improve CF practices^(^ ^41^ ^,^ ^42^ ^)^. Training should be provided in order for health-care professionals to deliver consistent information, as properly trained professionals can improve feeding practices and outcomes in children aged 6–24 months^(^ ^43^ ^)^. Given that eight of the nine studies that discussed advice providers highlighted the importance of family and friends, it seems advisable that interventions should target whole communities, not mothers alone.

### Strengths and limitations

The strengths of our systematic review are derived from searching a large number of databases utilizing very broad search strings, performing an updated search in June 2016, and having two reviewers undertake study selection, data extraction and quality assessment.

Key limitations include the exclusion of: (i) papers that focused solely on children over 2 years of age, where CFP described in their younger years may have been missed; (ii) papers published before the year 2000 at full-text review; and (iii) papers not published in English, which would have added to the diversity of CFP described. These limitations mean the review is potentially not exhaustive; however, we were able to screen a total of 45 712 abstracts across the four reviews. This is, to our knowledge, the most comprehensive review of the literature to date.

In several studies where there was overlap between children under and over 2 years of age and/or SA by Indian, Pakistani and Bangladeshi origin, CFP described and attributed to the whole study population may be incorrect. Furthermore, we did not assess the quantities of the foods used, only the frequency with which they appeared in the studies.

While we excluded interventional studies that may have described CFP in their study population, this is unlikely to be the primary focus of such studies and therefore unlikely to have affected our systematic review significantly.

## Conclusion

Despite adoption of the WHO IYCF guidelines, inadequate CFP remain in families across Pakistan. While Pakistan has made giant strides in decreasing child mortality over the last two decades, further work is required to improve CFP to reduce this further, as well as to tackle rates of wasting, stunting and underweight. The current systematic review has highlighted CFP and the factors that influence them, paving the way for development of evidence-based interventions.
